# Anisakidae and Anisakidosis: A Public Health Perspective

**DOI:** 10.3390/pathogens14030217

**Published:** 2025-02-22

**Authors:** Diana Nonković, Vanja Tešić, Vida Šimat, Svjetlana Karabuva, Alan Medić, Jerko Hrabar

**Affiliations:** 1 Department of Epidemiology, Teaching Institute of Public Health of Split-Dalmatia County, 21000 Split, Croatia; diana.nonkovic@nzjz-split.hr; 2 University Department of Health Studies, University of Split, 21000 Split, Croatia; 3 Department of Epidemiology, Teaching Institute of Public Health “Dr. Andrija Štampar”, 10000 Zagreb, Croatia; vanja.tesic@stampar.hr; 4 Department of Social Medicine and Epidemiology, Faculty of Medicine, University of Rijeka, 51000 Rijeka, Croatia; 5 University Department of Marine Studies, University of Split, 21000 Split, Croatia; vida@unist.hr; 6 Infectious Diseases Department, University Hospital of Split, 21000 Split, Croatia; skarabuva@kbsplit.hr; 7 Department of Epidemiology, Zadar Institute of Public Health, 23000 Zadar, Croatia; alan.medic@zjz.t-com.hr; 8 Department of Health Studies, University of Zadar, 23000 Zadar, Croatia; 9 Laboratory of Aquaculture, Institute of Oceanography and Fisheries, 21000 Split, Croatia

**Keywords:** *Anisakis*, *Phocanema*, *Contracaecum*, anisakidae, anisakidosis, public health

## Abstract

Fish and seafood are increasingly recognised as safe and nutritiously valuable foods of animal origin, being a source of about 17% of animal protein globally. Novel culinary trends encourage the consumption of raw or thermally lightly processed fishery products. At the same time, consumers prefer wild, fresh and whole fish over farmed or processed fish. However, the consumption of raw or undercooked fish and other marine organisms poses a risk of contracting parasitic infections, potentially representing a public health risk. Among the most common seafoodborne parasites are members of the Anisakidae family, especially the genus *Anisakis*, which can cause potentially detrimental effects to human health. These parasites are the causative agent of a zoonosis termed anisakidosis that is prevalent in countries with high per capita fish consumption. Although the number of annual clinical cases varies among countries and regions and is generally not high, sensitisation to this parasite in the general population seems to be considerably higher. Therefore, anisakidosis is still significantly underreported and misdiagnosed globally, making it a disease of rising public health concerns. To prevent infection and mitigate potential negative effects on human health, proper preventive measures such as gutting the fish, freezing or thermal processing are needed. Moreover, a holistic approach implementing One Health principles together with educational campaigns towards the general public and primary care physicians can extend the knowledge on the occurrence of these parasites in their natural hosts and the diagnosis and incidence of anisakidosis, with a final goal to minimize risks for human health and reducing costs for health systems.

## 1. Introduction

**Figure 1:**
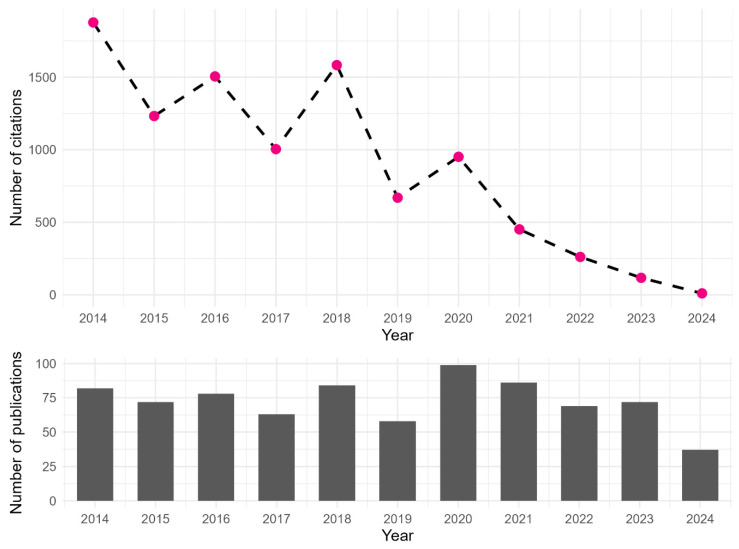
The number of publications (**bottom**) and citations (**top**) per year in the last decade (including 2024) in the Scopus database related to Anisakidae.

Fish and seafood are increasingly recognised as safe and highly nutritious foods of animal origin. Besides being an important source of high-quality protein, providing about 17% of animal protein at global level, they are a primary source of long-chain polyunsaturated fatty acids, including eicosapentaenoic (EPA) and docosahexaenoic (DHA) acids, but also of other essential components such as minerals, vitamins, peptides, collagen, etc. These features make fish and seafood a vital source of valuable nutrients for people worldwide and an essential component of a healthy and balanced diet. Fish ingredients have the ability to act as nutraceuticals and can help prevent diseases related to modern lifestyles, such as hypertension, obesity, cardiovascular disease, asthma and many others [1]. Therefore, consumer demand for fish and seafood is increasing, and global per capita consumption has risen to over 20 kg since 2018 [2]. Culinary trends encourage the consumption of raw or mildly preserved products, and consumers prefer wild, fresh and whole fish over farmed or processed fish [3]. Considering that raw and undercooked seafood may pose a risk of contracting parasitic infections, the consumption of such products could represent a public health risk [4]. According to the World Health Organization (WHO), foodborne diseases were responsible for 420,000 deaths in 2010 and a global disease burden of 33 million Disability-Adjusted Life Years (DALYs) [5]. The European Food Safety Authority (EFSA) considers *Anisakis* a major parasitological risk to humans associated with fish consumption [6,7] due to its ability to cause serious pathological conditions in humans as well its global distribution [8]. In addition, several species belonging to the genera *Phocanema* (a recently resurrected genus encompassing five species formerly belonging to the genus *Pseudoterranova* [9]) and *Contracaecum* in the family Anisakidae are also considered zoonotic and are recognised as causative agents of anisakidosis [7]. In Japan, anisakidosis has been the most common form of foodborne infection since 2017, its case numbers having exceeded those of *Campylobacter jejuni*/*coli* and *Norovirus* in infections [10]. Furthermore, the prioritisation of foodborne parasitic infections in Europe has listed the family Anisakidae among the top 10 foodborne parasites in every European region [11]. In fact, from January 2020 to date, there have been 108 notifications in the European Commission’s Rapid Alert System for Food and Feed (RASFF) regarding the presence of larvae of *Anisakis* spp. in fish and fish products, one notification regarding the presence of larvae of *Phocanema* spp. and a further eight notifications regarding the presence of undetermined Anisakidae larvae. Live or dead larvae were detected in fresh fish and various products from a number of economically important species [12]. From a public health perspective, the cosmopolitan zoonosis caused by members of the Anisakidae family, especially *Anisakis* spp., appears to be significantly underreported and underestimated in many countries or regions around the world, with significant gaps in knowledge about the epidemiological and ecological transmission mechanisms of these nematodes to humans [13]. The prevalence of specific human serum antibodies is a parameter that can indicate exposure to the parasite. Although it is evident that the rates of sensitisation to *Anisakis* spp. have increased in people around the world, to date, quality diagnostic criteria and laboratory algorithms for detecting anisakiasis have not been clearly established. Therefore, this disease continues to present an underreported health problem with severe clinical manifestations. When this parasite is present, it could mimic allergic or digestive disorders much more often than anticipated. Because of that, it is important to establish the correct diagnosis for possible public health interventions in populations with a high risk of exposure to the infection. It is also important to adapt current health services to better serve the population at risk for a more effective and less costly diagnostic screening [14]. A survey of family medicine doctors in Australia confirmed a low level of knowledge and awareness among health professionals about the risks to human health associated with parasites transmitted through seafood [15]. Another Australian study indicated a need for change in both medical and veterinary education as well as continuing professional education, especially for general physicians, to increase their knowledge on zoonoses [16]. According to the Scopus database, scientific interest in the Anisakidae has increased in the last decade (Figure 1); however, the average number of publications related to the impact of Anisakidae on public health over the past ten years is only 12.8/year. Furthermore, Shamsi [17] observed an increasing trend in the number of publications on Anisakidae from the 1940s to the end of the 2000s, with a sharp decline at the beginning of the following decade. Based on our search results, there did not appear to be a sharp decline until the end of the 2010s, but in the first half of the 2020s, a decline in the number of publications and especially citations on this topic can be seen (Figure 1).

**Figure 2:**
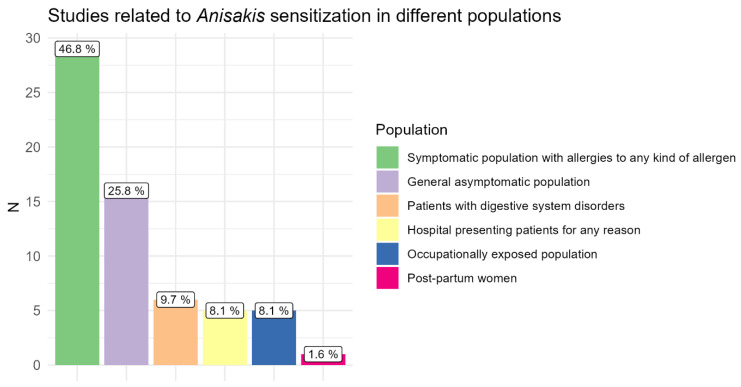
Percentage of studies related to *Anisakis* sensitisation in different populations included in systematic review by Mazzucco et al. [18] and published afterwards. Note that some studies included more than one population. General asymptomatic population refers to subjects not occupationally exposed to *Anisakis* allergens and without history of symptoms related to anisakidosis.

**Figure 3:**
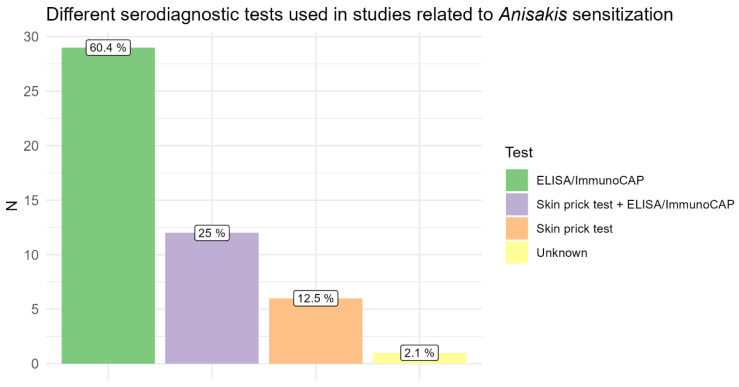
Percentage of different serodiagnostic tests used in studies related to *Anisakis* sensitisation.

Previously, Mazzucco et al. [18] identified 41 studies related to *Anisakis* sensitisation in a 21-year period in different population groups. Since then, we have identified only seven studies related to *Anisakis* sensitisation, either in allergic subjects or sensitised asymptomatic populations [19,20,21,22,23] or occupationally exposed populations [24,25]. The majority of studies (46.8%) included in the review and identified afterwards focused on symptomatic populations with allergies to any kind of allergen, while only 8.1% of published studies focused on occupationally exposed populations (Figure 2). Most used serodiagnostic tests were enzyme-linked immunosorbent assay (ELISA)/ImmunoCAP (60.4%), followed by ELISA/ImmunoCAP in combination with a skin prick test (SPT) (25%), while in 12.5% of studies diagnosis was based solely on a skin prick test (Figure 3).

The aim of this literature review is to provide a comprehensive overview of recent knowledge on Anisakidae and anisakidosis from a public health perspective, with a particular focus on *Anisakis* spp. as the most important genus and an increasing public health and food safety problem.

## 2. Biology of Family Anisakidae

**Figure 4:**
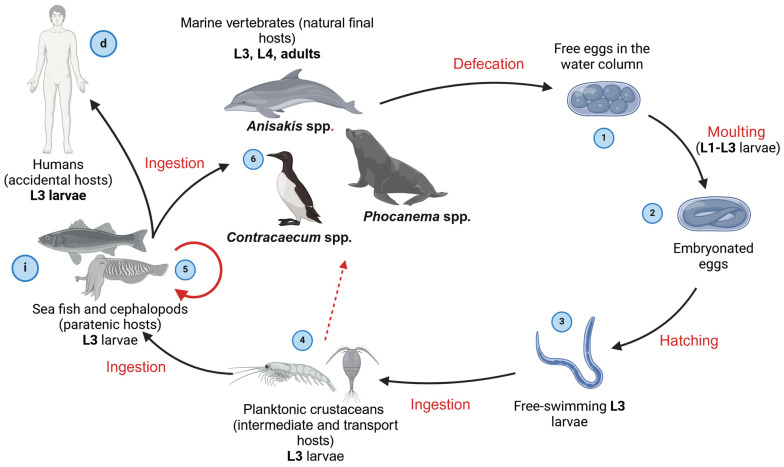
The general life cycle of anisakid nematodes. Gravid females release their eggs into the water column with the faeces of the final host (1), where they are embryonated (2). After two moults, the eggs hatch into free-swimming third-stage larvae (L3) (3), which are ingested by an intermediate host (crustacean) (4). In the intermediate host, the larvae migrate to the haemocoel and become infective. The intermediate host is then eaten by a paratenic host (fish, squid), in which the larvae spiralise in the visceral organs or the musculature (5). Small fish can be eaten by larger fish. In this case, the larvae repeat the process of migration and spiralisation (solid arrow) and can accumulate in large numbers. Alternatively, intermediate host can be eaten directly by the final host (e.g. baleen whales) (dashed arrow). The life cycle is completed when the intermediate/paratenic host is eaten by a final host, i.e., a toothed whale, a pinniped or a fish-eating bird, depending on the anisakid genus, in which the L3 develop into L4 (subadult) and sexually mature adults (6). In the life cycle of anisakids, humans can become accidental hosts after eating raw or lightly thermally processed seafood infected with anisakids L3. i—infective stage, d—diagnostic stage.

The family Anisakidae comprises, among others, the genera *Anisakis* Dujardin, 1845, *Phocanema* Myers, 1959 (*Pseudoterranova* Krabbe, 1878), and *Contracaecum* Railliet and Henry, 1913, whose members in adult stages parasitise the digestive tract of marine mammals and seabirds and can cause disease in humans [26]. These parasites share a general life cycle concept that utilises planktonic crustaceans as intermediate hosts, fish and cephalopods as paratenic (transport) hosts and marine mammals, i.e., cetaceans and pinnipeds, and fish-eating birds as definitive hosts (Figure 4). Due to high morphological similarities in larval stages, species differentiation is difficult and possible only in adult stages using a limited number of morphological traits, such as the features of the excretory system, alimentary canal, the number and distribution of male caudal papillae, the length of spicules and the position of the vulva [26,27]. In larval stages, however, identification is possible only at the genus level based on the shape and length of the ventriculus and the presence/absence of a mucron and boring tooth [26,28]. Therefore, proper species delineation relies on molecular techniques using several mitochondrial [29,30] and nuclear markers [31,32,33,34], which have proven useful in identifying species and their hybrid forms.

### 2.1. Genus Anisakis Dujardin, 1845

Historically, nine valid species, separated into four clades, were recognised within the genus *Anisakis*—clade 1: *A. pegreffii* Campana-Rouget and Biocca, 1955, *A. simplex* sensu stricto (s. s.) (Rudolphi, 1809), and *A. berlandi* Mattiucci, Cipriani, Webb, Paoletti, Marcer, Bellisario, Gibson and Nascetti, 2014; clade 2: *A. ziphidarum* Paggi, Nascetti, Webb, Mattiucci, Cianchi and Bullini, 1988, and *A. nascetti* Mattiucci, Paoletti and Webb, 2009; clade 3: *A. physeteris* (Baylis, 1923), *A. paggiae* (Mattiucci, Nascetti, Dailey, Webb, Barros, Cianchi and Bullini, 2005) and *A. brevispiculata* (Dolfus, 1966); and *A. typica* (Diesing, 1860) Baylis, 1920, as a separate lineage [13,27]. Recent systematics, however, placed the species of clade 3 in the restored genus *Skrjabinisakis* Mozgovoi, 1951 [35]. Furthermore, several additional genotypes closely related to *S. physeteris* and *A. typica*, respectively [13,36,37], have been reported; however, these have not been completely characterised genetically. Therefore, at the moment, it remains unknown whether these represent new, sibling species of the two aforementioned. Among the members of the genus *Anisakis*, only *A. pegreffii* and *A. simplex* (s. s.) are zoonotic, and the latter is considered more pathogenic, having a greater ability to penetrate fish musculature [38]. However, it should be taken into consideration that other species of the genus might be zoonotic, as species identification by genetic/molecular markers is not always performed. This is particularly valid for *Skrjabinisakis physeteris* and *S. paggiae*, until recently belonging to the genus *Anisakis*, for which infection potential in Wistar rats was demonstrated [39]. Nevertheless, there are no reported clinical cases relating anisakidosis with these two species; therefore, their true zoonotic potential and relevance for human health remains unknown. Considering this and the differences in infection potential that might exist between the congeners, proper species identification is necessary from a public health perspective.

**Figure 5:**
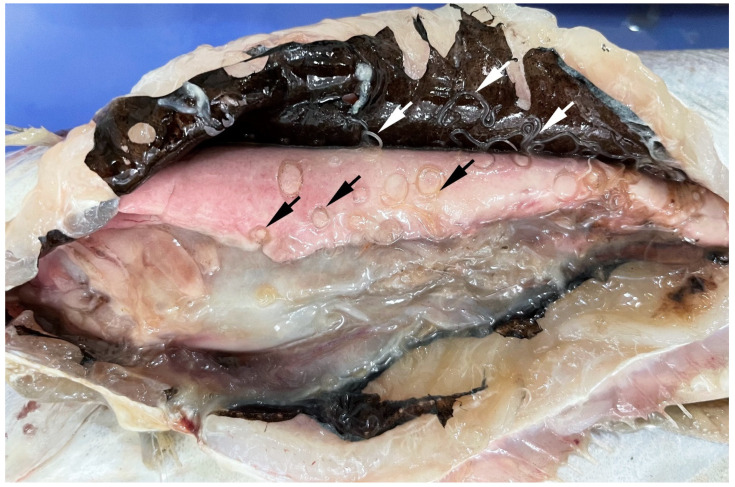
*Anisakis* spp. spiralised (black arrows) on visceral organs and actively moving in the visceral cavity (white arrows) of blue whiting, *Micromesitius poutassou*. (Photo credit: Jerko Hrabar).

*Anisakis* spp. has a heteroxenous life cycle involving more than one obligate host, with four larval stages (L1–L4) and an adult stage with fully developed reproductive organs [28,40]. These parasites utilise marine mammals, primarily toothed whales, as definitive hosts, fish and cephalopods as paratenic (transport) hosts and planktonic crustaceans as intermediate hosts [40] (Figure 4). Females disseminate fertilised eggs via cetacean faeces into the water column, where they become embryonated [41]. It is widely accepted that inside the egg, following two moults, a third-stage (L3) larva develops. A hatched L3 is ensheathed inside a thick L2 cuticle, which helps maintain the buoyancy of the L3 [42]. At this stage, the L3 is still not infective. However, a recent in vitro study [43] indicated that already L2, rather than L3, is the stage that hatches from the egg. Hatched larvae are eventually ingested by planktonic crustaceans, typically euphausiids and less frequently mysids, inside which they migrate to hemocoel, develop further and become infective [41,44,45]. Planktonic crustaceans are then preyed upon by fish and cephalopods, which serve as paratenic hosts [28,40]. Inside the paratenic hosts, *Anisakis* spp. migrate from the digestive tract into the visceral cavity or occasionally skeletal musculature, where they spiralise (Figure 5) and remain in a state of paratenesis until a paratenic host is ingested by a final host. Alternatively, smaller fish and cephalopods can be preyed upon by larger fish, in which case L3 repeats the migration from the digestive tract and spiralisation on visceral organs, resulting in the accumulation of a large number of parasites along trophic webs [28,46]. In the life cycle of *Anisakis* spp., humans can become accidental hosts following the consumption of raw or undercooked fishery products infected with L3, thus contracting a disease termed anisakidosis (anisakiasis) [47,48]. Recently, *Anisakis* L3 has been found in several unusual host species in the southern hemisphere [49]. While these represent interesting cases, whether these species can be considered as true hosts in which the larvae could develop further or represent a dead end for the larvae remains elusive, as no adult specimens have been found, nor has a transmission to natural final hosts been demonstrated.

**Figure 6:**
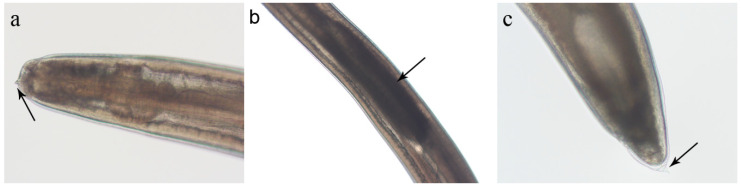
The morphology of the third larval stage of *Anisakis* spp. (L3), larval type I (sensu Berland, 1961). (**a**) The cephalic end with a small pyramidal boring tooth (arrow); (**b**) the anterior part with a dark-coloured ventriculus (arrow). Note that in living specimens the ventriculus appears as a white, barrel-shaped structure that is clearly visible to the naked eye; (**c**) a tail with a small spine (mucron) at the tip of the tail (arrow). (Picture credits: Jerko Hrabar).

Adults of the *A. simplex* sensu lato species complex, i.e., species belonging to clade 1, can be distinguished from other clades by the characteristics of the ventriculus, which is longer than it is wide and often sigmoid-shaped, and the male spicules, which are long, thin and unequal in length [27]. In contrast, the species of clade 1 cannot be distinguished from the species of clade 2 and the *A. typica* clade in the larval stage. The larvae are white or milky in colour, are up to 40 mm in length and exhibit the so-called type I larval morphology (sensu Berland, 1961), which is characterised by a long ventriculus and the presence of a mucron at the tail end (Figure 6) [50,51]. At the anterior end, there is a boring tooth near the opening of the excretory pore, and a nerve ring can be seen. The oesophagus consists of a proventriculus and a ventriculus, which appears white in live specimens, while the intestine narrows to open into the rectum, which opens into the anus [50]. Although some morphological features have been proposed between *A. simplex* (s. s.) and *A. pegreffii* [52], no valid diagnostic features have yet been found that allow for differentiation between the species with type I larvae [13].

**Figure 7:**
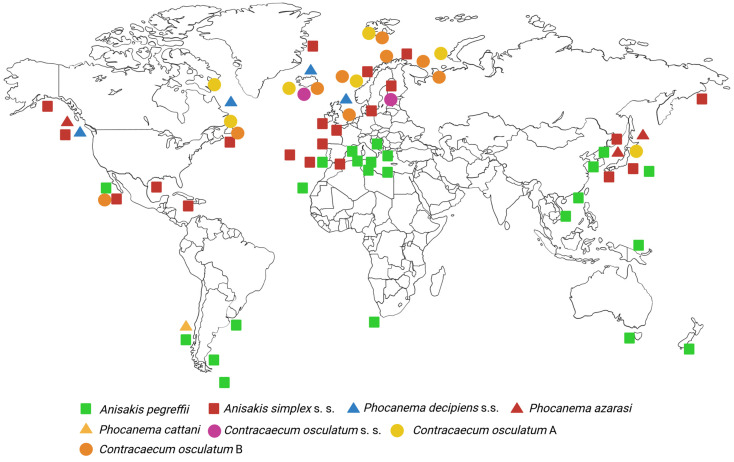
A world map showing the documented distribution of zoonotic species of Anisakidae in the period 1986–2018. Two Antarctic species of the *Contracaecum osculatum* complex, i.e., *C. osculatum* D and *C. osculatum* E, are not indicated on the map (adapted from [13,26]).

Members of the genus *Anisakis* have a global distribution and have been genetically confirmed from more than 40 species of final hosts and more than 160 species of paratenic hosts (Appendix A, Table A1) [13]. *A. simplex* is the Boreal species of the genus with a distribution range in colder waters ranging from approximately 35° N to the Arctic seas. On the other hand, *A. pegreffii* is the dominant species in the Mediterranean Sea but is also present in the Austral region, between approximately 30° S and 60° S (Figure 7) [13]. A recent meta-analysis of the global abundance of *Anisakis* revealed a significant 283-fold increase in *Anisakis* abundance in the period 1978–2015, indicating possible implications for marine mammal health, human health and fisheries’ profitability [53].

### 2.2. Genus Phocanema Myers, 1959

The genus *Phocanema* comprises at least six sibling species that formerly belonged to the genus *Pseudoterranova*, i.e., the *Pseudoterranova decipiens* species complex: *Ph. decipiens* (s. s.) (Krabbe, 1878) Myers, 1959, *Ph. azarasi* (Yamaguti and Arima, 1942) Bao, Giulietti, Levsen and Karlsbak, 2023, *Ph. krabbei* (Paggi, Mattiucci, Gibson, Berland, Nascetti, Cianchi and Bullini, 2000) Bao, Giulietti, Levsen and Krlsbak, 2023, *Ph. bulbosum* (Cobb, 1889) Bao, Giulietti, Levsen and Karlsbak, 2023, *Ph. cattani* (George-Nascimento and Urrutia, 2000) Bao, Giulietti, Levsen and Karlsbak, 2023, and *Ph. decipiens* sp. E Bullini, Arduino, Cianchi, Nascetti, D’Amelio, Mattiucci, Paggi, Orecchia, Plotz, Smith and Brattey, 1997 [9,26,54]. Of these, *Ph. decipiens* (s. s.), *Ph. azarasi* and *Ph. cattani* have been associated with infections in humans [55,56,57]. The members of the genus have a heteroxenic life cycle similar to that of the genus *Anisakis* (Figure 4), with some exceptions. Firstly, the life cycle of *Phocanema* spp. is associated with benthic and epibenthic habitats, in contrast to the pelagic life cycle of *Anisakis* spp., where hatched larvae attach to the substrate with their caudal end. Secondly, copepods of the orders Harpacticoida and Cyclopoida, rather than euphausiids and mysids, mainly serve as intermediate hosts, although several groups of benthic macroinvertebrates may also serve as intermediate hosts. Finally, pinnipeds, especially from the families Otariidae and Phocidae, serve as main definitive hosts [28,42,58].

**Figure 8:**
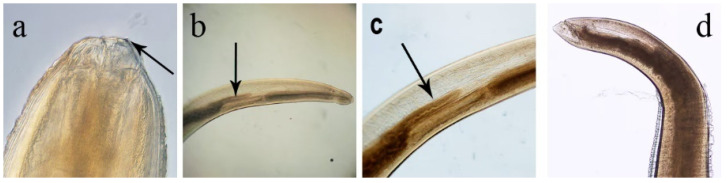
Morphology of *Phocanema* spp., third larval stage (L3). (**a**) Cephalic end with small pyramidal boring tooth (arrow); (**b**) anterior part with ventriculus and anteriorly projecting intestinal caecum (arrow); (**c**) detail of image in panel B showing intestinal caecum (arrow); (**d**) posterior end. (Picture credits: https://www.cdc.gov/dpdx/anisakiasis/ (accessed on 7 September 2024)).

The larvae of *Phocanema* spp. can be distinguished from other anisakids by their yellowish to reddish or brown colour and the presence of an intestinal caecum, which may be the same or different in size compared to the glandular ventriculus and protrudes forward (Figure 8) [51,59,60]. At the cephalic end, there is a small boring tooth, under which an excretory pore opens ventrally; the nerve ring is thinner than in *Anisakis* spp. At the tail end, the intestine narrows to open into the rectum, which opens into the anus surrounded by anal glands, while at the tip of the tail there is a short spine or mucron [51,60].

The species of the *Ph. decipiens* complex are also distributed worldwide, with four species occurring in the Northern Hemisphere (*Ph. decipiens* (s. s.), *Ph. krabbei*, *Ph. azarasi* and *Ph. bulbosa*) and being sympatric in certain areas and two species in the Southern Hemisphere (*Ph. cattani* and *Ph. decipiens* E) (Figure 6) [26,58]. These parasites have been detected by genetic markers in more than 10 species of definitive hosts and more than 20 species of paratenic hosts worldwide (Appendix A, Table A2) [26]. Of the three known zoonotic species, *Ph. decipiens* s. s. has a wide distribution, mainly in the Arctic and subarctic regions, in the coastal waters of the North Atlantic (off northern Europe and Iceland and eastern Canada, from Labrador to the Gulf of Maine) [54,61,62] and north-east Pacific [63]. *Ph. azarasi* occurs in Japanese waters [63,64] and in the eastern Pacific south of 30° N, probably due to the migration of its final host, the California sea lion (*Zalophus californianus*) [30]. Finally, *Ph. cattani* has been recorded in the south-east Pacific off the Chilean coast [59,65] and along the Patagonian coast of Argentina [66]. In contrast to *Anisakis* spp., no significant changes in the abundance of *Phocanema* spp. over a period of 37 years were detected in the most recent meta-analysis of global abundance [53].

### 2.3. Genus Contracaecum Railliet and Henry, 1912

The genus *Contracaecum* comprises about 50 nominal species that primarily parasitise pinnipeds and aquatic birds, with two complexes of sibling species, namely, *C. ogmorhini* sensu lato (s. l.) and *C. osculatum* (s. l.), comprising at least six sibling species [26,67,68,69]. The latter probably have zoonotic potential, as shown in experimentally infected pigs in which these nematodes caused eosinophilic granulomas in the stomach [70]), and in several documented cases of infection in humans after the consumption of raw fish [71,72]. These nematodes appear to have as complex a life cycle as members of other Anisakidae (Figure 4), including benthic and pelagic invertebrates (crustaceans and squid) and fish [73,74]. Similar to the eggs of *Phocanema* spp., the eggs of *Contracaecum* spp. are heavier than seawater and eventually rest on the seabed, where, after hatching, the larvae are most likely ingested by harpacticoid copepods [75] but may also be transferred vertically through the water column by other crustaceans and small fish that stay near the bottom during the day and feed pelagically at night [28]. The life cycle is completed as soon as an infected paratenic host is eaten by a seal (families Otariidae and Phocidae) or a bird (genera *Larus*, *Pelecanus*, *Phalacrocorax*), depending on the parasite species, in which the L3 develops into a fourth larval stage (subadult) and sexually mature adults.

**Figure 9:**
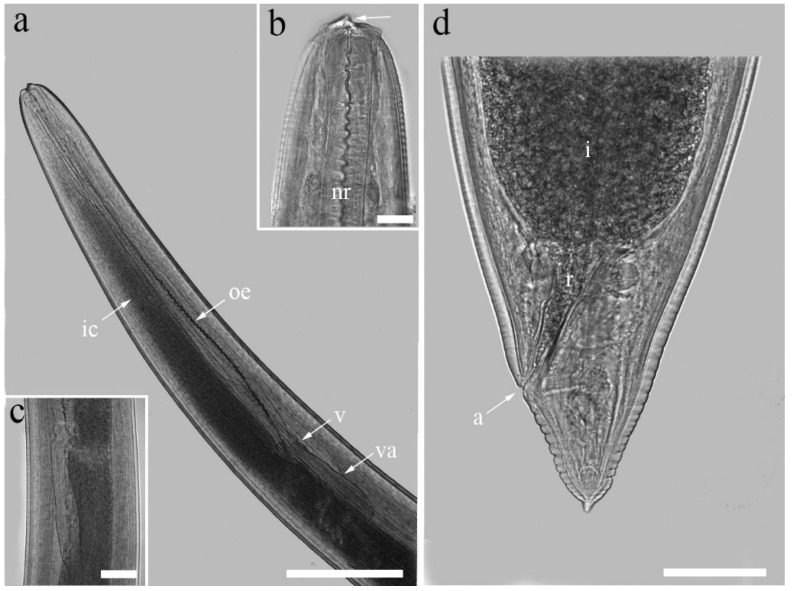
Morphology of *Contracaecum* spp., third larval stage (L3). (**a**) Cephalic end showing the oesophagus (oe), ventriculus (v), intestinal caecum (ic) and ventricular appendix (va); (**b**) detail of cephalic region showing boring tooth (arrow) and nerve ring (nr); (**c**) detail of ventriculus with clearly visible ventricular appendix running along dark-stained intestine; (**d**) posterior end with intestine (i) opening into rectum (r) and anus (a) and tail without spine (mucron). (Picture credits: [78]).

The larvae of *Contracaecum* can be distinguished from other anisakids by the shape of their digestive tract, which differs in the presence of an anteriorly directed intestinal caecum running along the preventriculus and a posteriorly directed ventricular appendix running along the intestine (Figure 9) [73,75,76]. At the cephalic end, there is a pyramidal boring tooth between the lateroventral lips and near the slit-like opening of the excretory pore [76]. At the caudal end, the intestine opens into the rectum, which opens into the anus, while the tail gradually tapers and terminates without a spine or mucron (Figure 9) [76,77]. The larvae in fish are about 7 to 30 mm long, greenish-brown in colour and lie curled up in capsules of irregular shape [77].

Species of the zoonotic *C. osculatum* complex are distributed in the Arctic and Antarctic, with *C. osculatum* A Nascetti, Cianchi, Mattiucci, D’Amelio, Orecchia, Paggi, Brattey, Berland, Smith and Biullini, 1993, *C. osculatum* B Nascetti, Cianchi, Mattiucci, D’Amelio, Orecchia, Paggi, Brattey, Berland, Smith and Biullini, 1993, and *C. osculatum* (s. s.) (Rudolphi, 1802) Baylis, 1920, occurring in the Arctic [79] and *C. osculatum* D Orecchia, Mattiucci, D’Amelio, Paggi, Plotz, Cianchi, Nascetti, Arduino and Bullini, 1994, and *C. osculatum* E Orecchia, Mattiucci, D’Amelio, Paggi, Plotz, Cianchi, Nascetti, Arduino and Bullini, 1994, in the Antarctic [69] (Figure 6). *C. osculatum* (s. s.) has so far been reported from the western Atlantic and the Pacific and is the only member of the *C. osculatum* complex that occurs in the Baltic Sea [26,67]. For a detailed distribution of the other species of this genus, the reader is referred to [26]. These nematodes have so far been genetically identified in 8 definitive hosts and 12 paratenic hosts (Appendix A, Table A3) [26].

## 3. Anisakidosis

According to the 1988 standardised nomenclature of parasitic zoonoses, anisakidosis is defined as an ichthyozoonosis caused by nematodes of the family Anisakidae [80]), while more specific terms are widely used depending on the genus to which the pathogen belongs: anisakiasis for the disease caused by *Anisakis* spp., pseudoterranovosis when the causative agent is *Phocanema* (*Pseudoterranova*) ssp. and contracaecosis when the disease is caused by members of the genus *Contracaecum* [80,81]. The disease is acquired accidentally following the consumption of raw or undercooked fish and cephalopods infected with live anisakid larvae [82]. The main sources of infection are marine fish species as the main ingredient of dishes such as sushi, ceviche, sashimi and other similar foods [82,83]. Generally, a single viable larva is sufficient to cause the disease, but cases of hyper infection with these parasites (N = 140) have been recorded [84].

### 3.1. Clinical Manifestations

Depending on the tissues in which the lesions are caused by the infecting larvae, patients may present with different symptoms. Anisakidosis caused by *Anisakis* spp. (anisakiasis) is the predominant form of the disease. Four clinical entities of anisakiasis are distinguished according to the localisation of the live or dead larvae in the body and the predominant symptoms: gastric, intestinal, ectopic and (gastro)allergic anisakiasis [4,83,85]. The presence of elevated titre of anti-*Anisakis* antibodies in healthy, asymptomatic, sensitised individuals is a possible fifth form of anisakiasis in humans, although for now this form is insufficiently explained [86]. Asymptomatic infection normally occurs when the larvae stay inside the gastrointestinal lumen without any adverse impact on the health of the host [87]. Both gastric and intestinal anisakidosis, collectively termed invasive anisakidosis, occur following the migration of larvae through the gastrointestinal wall and are associated with oedema and congestion, with the larvae embedded in inflammatory cell infiltrates in the gastrointestinal mucosa [88]. Symptoms resulting from gastric infections seem to appear 1–8 h post-ingestion, whereas intestinal infection often manifests after 5–7 days. The penetration of gastric mucosa is characterised by abrupt upper abdominal pain accompanied by nausea, vomiting and slightly elevated body temperature [83,89]. The clinical cases of acute gastric anisakiasis are rare even in geographic regions where the consumption of raw seafood is habitual [90]. Intestinal anisakiasis is characterised by intermittent or constant abdominal pain, tenderness of the abdomen, diarrhoea and luminal stenosis due to mucosal oedema, which can result in intestinal obstruction and bloody stool. Some individuals can present with rare complications such as intestinal perforation, peritonitis, intussusception and pneumoperitoneum [83,87,91]. Ectopic (extragastrointestinal, heterologous) anisakidosis occurs in cases when larvae penetrate the gastrointestinal wall and migrate to one of the visceral organs such as the liver, spleen, ovaries or local lymph nodes [83,92,93,94]. The migrating larvae can cause tumourous formations in the stomach wall and, more rarely, in the intestinal wall [95,96,97]. In the chronic type of anisakidosis, these tumour-like formations are eosinophilic granulomas, which can be located submucosally [97,98] or in the muscularis externa [95]. In the acute or intermediate type of anisakidosis, these formations are known as vanishing tumours which resolve (disappear) after the physical removal of the invading larvae [97,99,100,101,102]. The migrating larvae can also cause nodules on internal organs, which can mimic certain malignancies such as liver cancer, which can be seen on clinical images [92,103]. Apart from being the causal agent of gastrointestinal pathology, *Anisakis* species are considered to be the only parasites in fishery products that are implicated in allergic reactions [83,104,105]. Allergic anisakiasis is the most common clinical form of anisakidosis and will be discussed in more detail in a separate section.

Anisakidosis (pseudoterranovosis) caused by *Phocanema* spp. is rarer, usually affects only the stomach and is generally milder than anisakidosis caused by *Anisakis* spp. [83]. Infections with *Phocanema* spp. are often asymptomatic and do not lead to tissue invasion. However, they can lead to medical attention when live or dead larvae are coughed up, vomited or excreted with faeces [83,106,107]. Cough is usually the primary symptom of non-invasive pseudoterranovosis, although infected individuals may also have other symptoms such as sputum production, pharyngeal pain, nausea and anal and nasal pruritus. The excretion of the worms through coughing can occur up to 7 days after eating raw, infected fish [106]. People with non-invasive pseudoterranovosis may also experience the so-called “tingling throat syndrome” when the worms crawl up the oesophagus into the oropharynx [48,57,108]. Gastric (invasive) pseudoterranovosis was first described in Japan in 1972, when several cases of infection were found during gastroscopy of patients admitted to hospitals complaining of severe epigastric pain several hours after a fish meal [109,110]. In general, the symptoms of gastric pseudoterranovosis do not differ significantly from the symptoms of gastric anisakidosis. The only notable difference is that *Anisakis* spp. can invade virtually any part of the stomach, whereas the larvae of *Phocanema* spp. almost always invade the gastric mucosa of the greater curvature, where they can cause various types of lesions, including redness, swelling with subepithelial oedema, cellular infiltration and proliferation of lymphoid follicles [55,109,110,111,112]. In rare cases, *Phocanema* spp. can penetrate the gastric wall, end up on visceral organs causing extragastrointestinal anisakidosis and mimic different, more severe conditions such as liver cancer [113] or strangulated inguinal hernia [56]. Other unusual locations of infections were also documented, including the palatine tonsil [114] and larynx [115]. In contrast to *Anisakis* spp. which occurs in the L3 stage in infected humans, *Phocanema* spp. has often been extracted from infected individuals in the fourth larval stage (L4) [55,114,115,116].

Anisakidosis due to *Contracaecum* spp. (contracaecosis) is the rarest form of anisakidosis; therefore, scarce data are available on its clinical presentation. However, from an Australian case of infection with this parasite, which also presented with the digestive tract symptoms in the form of vomiting, diarrhoea and abdominal pain [72], it is likely that the symptoms are unspecific and do not differ much from other forms of anisakidosis. Although tissue penetration did not occur in the aforementioned case, experimental infections of different mammals show the potential of *Contracaecum* to penetrate the gastrointestinal wall and cause pathologic changes at the attachment site [70,117].

### 3.2. Diagnosis

The clinical diagnosis of anisakidosis is generally based on examination of the presenting symptoms and patient history—particularly dietary habits. Therefore, the first step for providing the correct diagnosis of gastrointestinal anisakidosis is collecting correct details of anamnestic data—clinical history, especially epidemiological data. An accurate diagnosis is crucial because clinical presentation may determine the clinical management of patients. The onset of intestinal anisakidosis (anisakiasis) varies from 1 to 7 days after the ingestion of the raw fish and differs from that of gastric anisakidosis, which develops symptoms a few hours after the ingestion of raw fish. However, there are cases in which the patients forget the food they ate, and thus, one should be very careful in collecting clinical history details. A precise reading of the findings of the clinical images is the second step that is required for making a correct diagnosis of intestinal anisakiasis [89,118].

In gastric anisakidosis, physical examination can reveal moderate tenderness in the epigastric region, which can be misdiagnosed as a peptic ulcer. An upper endoscopy can accurately detect gastric anisakidosis with filiform larva firmly adhering to inflamed and swollen mucosa and its anterior extremity embedded in the stomach mucosa [89,90]. The final diagnosis can be made after a parasitological examination of the extracted larvae to determine the genus to which the pathogen belongs based on the morphological characteristics described above.

Cases of intestinal anisakidosis are not only rare, but their diagnosis is rather challenging due to the non-specific symptoms and often unreachable areas by standard endoscopic examination, although capsule endoscopy or double-balloon endoscopy can be performed in some institutions [89]. Therefore, intestinal anisakidosis can be misdiagnosed as appendicitis, peritonitis, intestinal obstruction or acute ciliopathy [89,119]. However, exact diagnosis can be confirmed by exploratory laparotomy, but this procedure is invasive and can cause multiple complications. A computerised tomography scan of the abdomen shows localised swelling and oedema of the small bowel, the dilatation of the intestine with fluid collection on the oral side of the lesion and the collection of ascites [89,90]. Ultrasound of the abdomen shows unspecific findings: marked local oedema of Kerckring’s fold, known as the corn sign, the dilatation of the oral portion of the small intestine with fluid accumulation and the accumulation of ascites [89,90].

Laboratory abnormalities in gastrointestinal anisakidosis usually include mild to severe leucocytosis and elevated serum levels of inflammatory markers, such as C-reactive protein. However, peripheral eosinophilia is rare and not constant, especially at the beginning of clinical manifestations [8,119].

**Figure 10:**
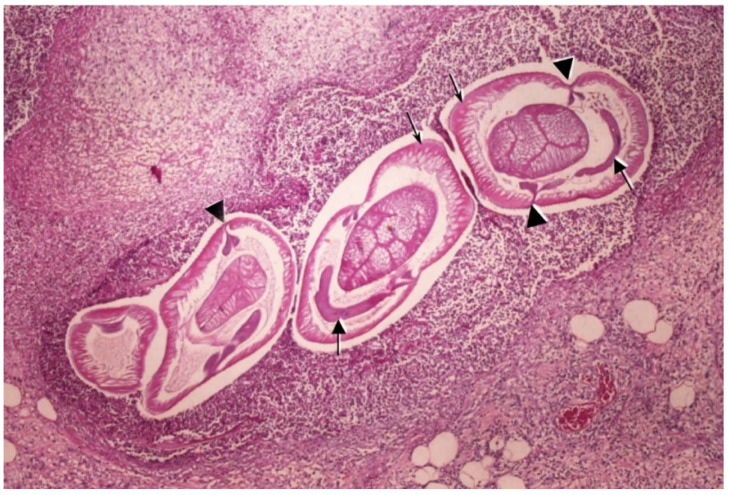
A histological section of a paraffin-embedded eosinophilic granuloma with four cross-sections of the third larval stage (L3) of *Anisakis* spp. Polymyarian muscle cells (thin arrows) are divided into four quadrants by two Y-shaped lateral chords (arrowheads). Two cross-sections show a banana-shaped excretory gland cell (Renette cell) (thick arrows), which is located ventrally to the intestine. (Picture credits: [123]).

The only method of diagnosing anisakidosis apart from endoscopy is immunological examination with serological tests to detect anti-Anisakis IgA, IgG and IgE antibodies [18]. Commonly used are the skin prick test, Western blot and ImmunoCAP, which have good sensitivity but low specificity because they use crude parasite extracts, which can lead to antigenic cross-reactivity with other related nematode species or other common allergens [120]. However, indirect enzyme-linked immunosorbent assay (ELISA) using recombinant Ani s 1 and Ani s 7 allergens as the target is now considered a gold standard in serological diagnosis of anisakidosis [121]. In cases where *larva migrans* causes nodules on visceral organs and surgical resection is performed, the diagnosis can be made on the basis of pathohistological findings of paraffin-embedded specimens, in which cross-sections of larvae with a characteristic histological appearance and surrounded by abundant mixed cellular infiltrate are frequently detected. These characteristics include large polymyarian muscle cells divided into four quadrants by Y-shaped lateral cords, columnar epithelial cells and a circular gut with a triangular lumen. Depending on the section, a large banana-shaped excretory gland cell (Renette cell) can also be seen (Figure 10). In certain cases where the larva is degraded and no morphological features can be distinguished in the histological specimens or if the exact species of the pathogen is to be determined, DNA extraction from paraffin-embedded tissues or extracted larvae and subsequent amplification and sequencing of one of the diagnostic markers (mitochondrial, nuclear) can be performed to confirm the diagnosis of anisakidosis and the pathogen [56,57,95,113,122]. Nevertheless, it should be noted that in such cases the accuracy of diagnosis, i.e., pathogen identification, will greatly depend on the quality of sequences available. As the sequences deposited in some public databases are not curated, this poses a possibility of misidentification and issues with the metadata related to a specific database entry.

### 3.3. Treatment

The choice of treatment in the diagnosis of anisakidosis depends largely on the form of the disease and the findings on clinical examination, particularly clinical imaging if an intestinal form is suspected, and ultimately varies from case to case. In the case of acute gastric anisakidosis, the treatment of choice is the extraction of larvae via upper endoscopy. The physical removal of anisakid larva adhering to the gastric wall using an endoscope (e.g., Roth net) is often curative without further need for pharmacological treatment [55,84,124,125,126]. However, due to the rare occurrence of the disease and the lack of experience of the staff performing the endoscopy, the larvae can easily be overlooked when they are hidden between the oedematous folds of the gastric mucosa [127]. Narrow-band imaging endoscopy and the use of L-menthol have been reported to facilitate the removal of larvae during endoscopy when the gastric mucosa is oedematous and erythematous and the larvae are vigorously moving, to increase contrast and inhibit gastrointestinal spasm and larval movement, respectively [128]. In cases where the larva has invaded deep into the gastric wall and its removal is not possible with standard biopsy forceps, jumbo forces which enable large tissue biopsies can be used to successfully remove the invading larva [122]. Intestinal anisakidosis is not only more difficult to diagnose but also more difficult to treat. Depending on which part of the intestine is affected, different therapeutic approaches have been reported. In several cases, intestinal anisakidosis, i.e., small bowel obstruction, has been successfully treated with conservative therapy, usually involving the administration of parenteral or intravenous prednisolone with or without concomitant administration of antihistamines [129,130]. Conservative therapy, including intra-venous fluid administration and analgesics, usually improves the clinical state and the symptoms associated with the acute inflammatory process resolve after one week [131]. The more invasive therapeutic procedures for intestinal anisakidosis include double-balloon enteroscopy for jejunal anisakidosis [132] or colonoscopy if the terminal ileum or colon are affected [133,134,135,136,137]. In many cases, however, especially when complications are encountered, surgical resection and manual anastomosis of the resected intestine is performed as part of an exploratory laparotomy or laparoscopy to remove masses or severe intestinal obstruction seen in clinical images [88,126,138,139]. In cases of strangulation or severe long segmental stenosis of the intestine, urgent surgical therapy is obligatory [87]. In addition, surgical resection is often indicated in ectopic anisakidosis due to the suspicion of malignancy before the final diagnosis is made on the basis of the histopathological findings of the resected samples [56,92,95,103,113]. This is probably the reason why, to our knowledge, there are no reports of the use of conservative therapy for ectopic anisakidosis. In addition, the diagnosis of ectopic anisakidosis is often only made at an advanced stage, when the larva has already decomposed and formed tumourous structures or abscesses, so the effectiveness of conservative therapy is questionable. The symptomatic treatment of acute allergic reactions in (gastro)allergic anisakiasis is no different from that for food allergies [104]. In patients with mild allergic reactions such as urticaria, the administration of second-generation antihistamines is usually sufficient to control the symptoms [104,140,141,142]. In severe cases or in patients with angioedema, immunomodulatory drugs such as systemic corticosteroids may be necessary [104,140]. In extreme cases, i.e., anaphylaxis, intramuscular adrenaline is indicated, and patients should be prescribed an adrenaline autoinjector [140,141].

The use of anthelmintics, mostly albendazole, is somewhat controversial. Although the U.S. Centres for Disease Control and Prevention lists 400 mg of albendazole twice daily for 6 to 21 days as a potential therapy in cases with presumptive diagnosis (https://www.cdc.gov/anisakiasis/hcp/clinical-care/ (accessed on 7 September 2024)), it is not approved by the U.S. Food and Drugs Administration for this indication. In vitro studies show that albendazole is effective in killing *Anisakis* spp. larvae [143], and it has been used in several documented cases of anisakidosis with subsequent resolution of symptoms [144,145,146,147,148]. Nevertheless, its efficacy in the treatment of *Anisakis* spp. infections remains elusive. In light of that, in the last few years, the research focus has shifted on evaluating different natural products for the treatment of anisakidosis. The administration of a medicine containing wood creosote (Seirogan) was successful in treating gastric anisakidosis [149], and wood creosote also potently inhibited larval movement in vitro [150]. In addition, the nematicidal activity of various natural products such as phytochemicals, essential oils and their components has been tested. Indeed, many have shown nematicidal potential against *Anisakis*: extracts and essential oils of ginger (*Zingiber officinale*), thyme (*Thymus vulgaris*), tea tree (*Melaleuca alternifolia*), nutmeg (*Myristica fragans*) and oregano (*Origanum vulgare*) (summarised in [151]). Recently, Mladineo et al. [152] compared two widely used anthelmintics, levamisole and abamectin, and two natural monoterpenes, farnesol and nerolidol, with different nematicidal activity and showed that ATP-binding cassette proteins (ABC proteins) are involved in the ejection of anthelmintics. In addition, Trumbić et al. [153] showed that in actively invading larvae, a list of the differentially expressed genes contains UDP-glucosyltransferases, which are involved in the excretion of toxic lipophilic and electrophilic metabolites. These metabolites are associated with ageing and reduced longevity in *Caenorhabditis elegans* and probably play a role in the persistence of *Anisakis* larvae and likely account for the variable efficacy of administered drugs.

## 4. *Anisakis* spp., a Hidden Food Allergen

Although allergic symptomatology is not common in helminth infections, it is more often observed in certain zoonotic infections such as anisakiasis [154]. *Anisakis* spp. is considered one of the most important hidden food allergens and is responsible for about 10% of previously unexplained idiopathic anaphylaxis as well as a significant number of urticaria in the adult population [155].

In patients with allergic reactions to *Anisakis* spp., an IgE-mediated immune response is triggered, resulting in different symptoms, ranging from urticaria and angioedema to anaphylaxis in extreme cases. Specific anti-*Anisakis* IgE antibodies can be detected in sera of sensitised subjects even after several years [156]. High intensity of *Anisakis* infection can significantly stimulate the activation of T-regulatory cells as well as the production of certain cytokines, primarily interleukin-10, and thus show a protective effect, while low levels of infection by stimulating T-helper 2 response worsens the body’s allergic response [157]. This hypothesis may explain why *Anisakis* spp. causes allergies in humans. Apart from the fact that the parasite is not adapted to coexist with humans, the number of larvae that are introduced into the body by eating fish is usually small, and the infection itself is of a transient nature [158]. Allergic reactions following the consumption of seafood containing the parasite or its antigens can be caused by either somatic antigens or excretory–secretory (ES) products of the infective larvae. So far, the existence of 14 *A. simplex* allergens (termed Ani s 1 to 14) (Table 1) has been proven, mostly belonging to the group of ES products [159]. The serological laboratory analyses of blood samples from patients with allergic manifestations of anisakidosis showed the most common reaction to Ani s 1, 5 and 7, considered to be the main allergens of the parasite. The Ani s 1 was identified in 85% of patients who developed clinical symptoms as a result of previous parasite infection [160]. Ani s 7 is an ES antigen, detected in the acute phase in 100% of patients with *Anisakis* allergies [161]. Due to its complexity, the relationship between the clinical presentation of allergy and sensitisation with multiple allergens in the case of *Anisakis* spp. has not been fully resolved. Based on the results of their research, Daschner et al. [162] observed the probable existence of different atopic phenotypes.

*A. simplex* allergy following the consumption of mackerel was first described in 1990 in Japan. All of the 11 patients with urticaria had a positive reaction to *A. simplex* L3 antigens, and none of the patients reacted to mackerel tissue antigens [174]. Sensitised patients can react to *Anisakis* spp. antigens (allergens), not only when these are present in food but also in small amounts of antigens due to other routes of exposure such as skin contact or the inhalation of allergens [86]. Moreover, although the consumption of seafood infected with *Anisakis* is the most common cause of allergic manifestations, the occurrence of allergic symptoms in eight patients after the consumption of chicken was described and related to the high proportion of fish meal used for poultry feed, which was likely contaminated with the parasite antigens [175]. Therefore, the immune response to *Anisakis* spp. should be reviewed from two aspects: the presence of the L3 larva (parasite) itself in the organism and the allergenic form [105].

Heat treatment or the freezing of raw fish as recommended procedures for the devitalisation of infective larvae have no effect on the allergenic potential of parasites. *Anisakis* spp. allergens are extremely resistant to high and low temperatures [176,177,178,179]. Therefore, there is still no preventive measure that would fully protect the consumer from the possibility of developing an allergic reaction to this parasite [6]. Thermostable parasite antigens cause the same cellular response as the total protein extract of *A. simplex*, confirming the risk even when consuming thermally processed food infected with the parasite [158].

## 5. Epidemiology of Anisakidosis

The first probable case of human infection by members of Anisakidae family was recorded in 1876 by Leuckhart [180]. In 1960, after several people consumed salted herring in the Netherlands, Van Thiel noticed and described the “very unusual finding” of a sea worm (herring worm) in the centre of an eosinophilic granuloma in a patient with acute abdominal pain [181]. As mentioned earlier, humans are not an integral part of the biological life cycle of Anisakidae, except as an accidental host. Larvae do not turn into adults in humans, so they do not have the ability to reproduce. Also, there is no interhuman transmission of the parasite [4]. The effects of environmental changes on foodborne parasites, including Anisakidae, should be studied and managed considering their complexity. Therefore, it is important that biologists, ecologists, physicians and veterinarians assess Anisakidae and anisakidosis under the one health concept and recognise human, domestic and wild animal and plant health as closely linked and interdependent [182]. The species predominantly associated with anisakidosis are *Anisakis simplex* and *A. pegreffii*. According to the EFSA, there were 20,000 estimated cases of anisakidosis worldwide prior to 2010, of which 90% were reported in Japan, a country with the highest reported annual incidence of 2000–3000 cases [6,47]. In Europe, the highest number of cases has been reported in countries with a high per capita consumption of fish and fisheries’ products, such as Spain, Italy, France, the Netherlands, the UK and Germany [4,6,8,26]. Except for Antarctica, anisakidosis has been reported from all continents [13]. Orphanet’s rare disease epidemiological data estimate worldwide incidence of anisakidosis in 2022 to be 0.32/100,000 (https://www.orpha.net/orphacom/cahiers/docs/GB/Prevalence_of_rare_diseases_by_alphabetical_list.pdf (accessed on 19 March 2023)).

Despite rising public health concerns, due to unspecific symptoms resembling other clinical entities, anisakidosis is still misdiagnosed and highly underreported. Epidemiological studies dealing with the incidence of anisakidosis in the general population are scarce and non-standardised, preventing comparison between studies and different geographical regions. Moreover, anti-*Anisakis* IgE antibodies can be present in the serum of sensitised individuals for several years and boosted by repeated exposure to *Anisakis* allergens [156], thus preventing the determination of primo-infection. A quantitative risk assessment analysis based on the consumption of anchovies in Spain estimated the number of annual *Anisakis* infections requiring medical attention to be between 7770 and 8320 [183], while officially reported annual cases for Spain range between 3.87 and 19.3 per 100,000 inhabitants. Two epidemiological studies based on national hospital discharge reports (HDRs) in Italy and Spain, respectively, reported much a lower number of confirmed cases, ranging from 370 cases between 2005 and 2015 in Italy [119] to 2471 cases in Spain between 1997 and 2015 [184]. The latter study also estimated the possible incidence of anisakidosis due to *Anisakis* to be as high as 20,978 cases annually. Such discrepancies between the reported and projected number of annual cases underline the fact that anisakidosis is still highly underreported and the importance of correct diagnosis. Nevertheless, as much as plausible, the results of the model should be interpreted with caution, as these will only be close to the real situation to the extent that the input data are valid. Interestingly, in France, only 37 cases have been confirmed by all parasitological laboratories of university hospitals (ANOFEL network) in a five-year period [185], while in Croatia a single case has been confirmed at pathohistological examination and molecular identification of *Anisakis* larvae [95], with another suspected case without the detection of the parasite [186]. Seroepidemiological studies targeting the general asymptomatic population [187,188,189,190,191,192,193] and different occupationally exposed populations [24,157,194] reported variable numbers of seropositive subjects, depending on the number of tested individuals, geographical regions, serological test used (skin prick test, ELISA, ImmunoCAP) and type and number of target allergens used, confirming that most seropositive subjects likely exhibited no significant clinical symptoms that would require hospitalisation. In general, in asymptomatic populations, anti-*Anisakis* seroprevalence ranged from 0% (0.4%) in Norway (ELISA vs. ImmunoCAP) to 22.1% in Spain [188], although the latter study tested only 77 individuals. In occupationally exposed populations (symptomatic and asymptomatic), i.e., fishermen/fishmongers and fish-processing industry workers, seroprevalence ranged from 1.8% (3.6% with *Anisakis* crude extract) when tested by indirect ELISA [24] up to 46.4% when tested by the skin prick test [194]. It is considered that employees in the fish industry may have a significant occupational risk of exposure to *Anisakis* spp. [195], which is in line with the so-far-reported higher prevalence compared to the general population. In symptomatic populations with allergies to any kind of allergen, the numbers of *Anisakis* seropositive individuals are much higher, reaching prevalence of 50–60% or even higher [18]. However, a recent systematic review reported that the highest prevalence of allergic anisakidosis was recorded in Portugal and Norway, ranging between 18.45% and 22.50% [196]. The authors did not seem to discriminate between different populations, resulting in considerably higher seroprevalence than when considering a specific population.

Anisakidosis caused by *Phocanema* spp. (pseudoterranovosis) is reported much less frequently than anisakidosis caused by *Anisakis* spp. (anisakiasis). However, due to the generally milder clinical presentation compared to anisakiasis, it is likely that pseudoterranovosis is also significantly underreported. Furthermore, there are much fewer epidemiological data available for pseudoterranovosis, which makes it difficult to discuss its actual incidence and burden of disease. Anisakidosis caused by *Phocanema* is generally rare in Europe but relatively frequent in Japan, Korea, North America and Chile. In Japan, a country with the highest incidence of anisakiasis, pseudoterranovosis also appears to be relatively widespread. It accounts for about 11% of all cases of anisakidosis, and 160 cases had been reported by the end of 1980s. All cases were caused by *Ph*. (*Pseudoterranova*) *decipiens* [111], although it is likely that other species from the *decipiens* complex were also involved, as reported for *Ph. azarasi*, which was molecularly identified in one case of infection in Japan [116]. By the mid-1990s, 796 cases of pseudoterranovosis had already been reported in Japan [197]. In South Korea, 660 cases of infection with *Phocanema* were reported by 2015, which corresponds to 11.8% of reported cases of anisakidosis [125]. Although the species causing the infections described in the review were considered to be *Ph. decipiens*, only recently have several cases been confirmed by molecular identification to be caused by *Ph. decipiens* s. s. [198]. In Chile, seven cases of anisakidosis caused by *Ph. decipiens* were diagnosed between 1997 and 1999 [106], while four further cases of infection with *Ph. cattani* were detected between 2012 and 2014 [108]. In addition, in South America, human infections with *Ph. cattani* and *Ph. decipiens* have been reported in Argentina [57] and in Peru [199], respectively. In Europe, the highest number of cases to date has been reported in Iceland, where 16 cases caused by *Ph. decipiens* were reported between 2004 and 2020. In contrast to Japan and other countries, *Ph. decipiens* accounted for 89 percent of all reported anisakidosis cases in Iceland, while *Anisakis* spp. was reported in only 11 percent (*n* = 2) of cases [107]. In recent years, cases of *Ph. decipiens* (s. s.), which have been molecularly identified, have also been reported in Italy [200], France [201] and Denmark [202], although in the last case with the unusual nasal location of the larvae.

Anisakidosis caused by *Contracaecum* is the rarest form of illness caused by the different anisakidae species. To date, only three cases of human infection have been reported, two in Japan [71] and one in Australia [72]. In the Japanese cases, the infecting larvae are referred to as *C. osculatum* (s. l.), but in the Australian case the larvae have not been identified to the species level.

## 6. Food Safety Considerations and Prevention of Anisakidosis

There is an inextricable link between food safety and nutrition, which has been of the most importance in times of climate change, food chain globalisation and modernisation of culinary techniques and food choices [203]. Food safety and food security are areas that urgently need the One Health approach, at all levels of academia, governance, industry, policy and research, because of the inseparable interconnectedness of animal, environmental, human, plant and planet health [204]. Contaminated food is a growing public health issue, which brings substantial economic losses, burdens health care systems and leads to malnutrition, especially in sensitive, young and elderly populations [205]. The harmful parasites such as Anisakidae nematodes are among biological hazards related to fish and seafood [206]. The incidence of anisakidosis has increased in recent decades following the trend toward the consumption of raw and mildly preserved (cold-marinated, cold-smoked, lightly salted) fish and seafood, representing a concern for producers, consumers and official control authorities. To prevent infection with live Anisakidae larvae, several options are mandatory or recommended for producers and consumers (Table 2). For mild treatments that do not ensure the killing of the parasite, it is critical to freeze the fish at −20 °C for no less 24 h or −35 °C for no less than 15 h in all parts of the product [207], while heating over 60 °C for at least 10 min is considered sufficient to kill larvae in 3 cm-thick fillets, thus avoiding infection with the live larvae [105]. The U.S. Food and Drug Administration (FDA) recommends that seafood intended for raw consumption should be regularly frozen at − 20 °C or below for 7 days or blast-frozen at − 35 °C or below for 15 h [208]. Recently, it has been shown that the type of fish product (fillet or whole fish) and the performance of the freezer must be considered to ensure effective larval kill [209]. These measures may be applied to raw material or finished products before they are placed on the market, questioning whether domestic freezers meet the cooling capacity and temperature regime criteria to comply with the regulations. Improper application of freezing therefore poses a high risk to consumers and small businesses [210].

Besides freezing and thermal treatment, other processing techniques showed no ability to kill L3 larvae in a short time. In chemical conservation processes such as marinating, salting and conservation by use of chemical or natural extracts, *Anisakis* larvae have shown strong resistance and high survival rate. According to the EFSA report [6], salting procedures using NaCl at a concentration of 5–9% can kill *A. simplex* larvae within 6 to >17 weeks, while dry salting procedures kill larvae in approximately 20 days [212]. Acid treatments and exposure to different solutions used in fish marinating (salt and sugar solutions of different concentrations, lemon juice, acetic acid, different vinegar solutions) also require at least 48 h to several months to kill larvae [212]. At high concentrations, the synergistic effect of acetic acid (10%) and salt (12%) may be more effective, requiring 5–28 days to kill larvae [213]. Recently, pulsed electric field (PEF) treatment was evaluated for the inactivation of *Anisakis* larvae in fishery products, showing strong potential but also dependence on PEF treatment parameters [214].

As an alternative to freezing and heat treatment procedures, the anisakicidal effect of plant extracts and essential oils has been reported. Both in vitro tests and tests on anchovy fillets showed the efficacy of cinnamon and rosemary oils against *Anisakis* larvae [215]. Similarly, oils flavoured with cumin and a mixture of parsley, garlic and lemon devitalised *Anisakis* larvae both in vitro and ex vivo within 24 h and after 8 and 10 days of exposure, respectively [216]. *Tagetes minuta* essential oil (1.0% and 5.0% *v*/*v*) also showed activity against *Anisakis* L3 in saline solution and industrial marinating solution, inactivating the parasite after 2 and 4 h, respectively [217]. The anisakicidal effect of R (+) limonene (LMN) was tested in marinated anchovy fillets at concentrations of 0.5%, 1% and 5% during the marinating process and storage in sunflower seed oil. The addition of 1% LMN during marinating and in the finished product packed in sunflower oil completely inactivated the larvae for 7 days without affecting the product’s sensory attributes [218]. These natural options, which show larvicidal potential, can be considered as treatments in the industrial process to reduce the risk of anisakiasis in humans.

**Figure 11:**
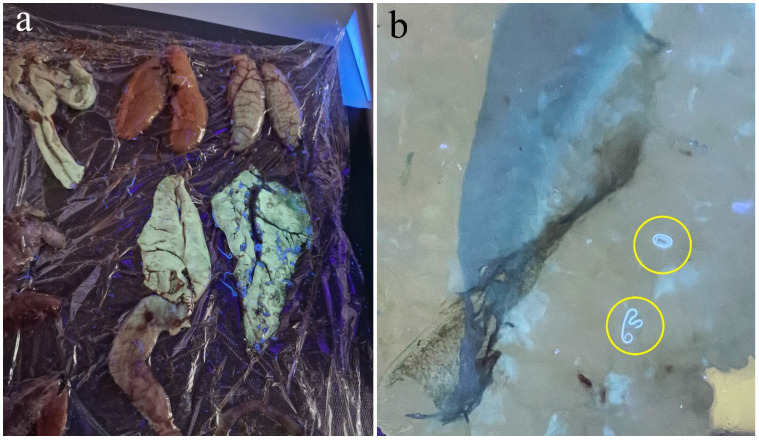
Blue fluorescence third larval stage of *Anisakis* spp. exposed to UV light. (**a**) *Anisakis* spp. larvae on fish gonads; (**b**) larvae (yellow circles) in pressed and frozen fish fillets (picture credits: Vida Šimat and Jerko Hrabar).

Despite all the above-mentioned measures, food business operators must visually inspect fishery products, and fish infested with parasites should not be placed on the market [219]. There are several methods recommended for anisakid inspection in the fishing industry, including visual inspection [220], candling [221], the most commonly used hydraulic pressing and inspection under a UV light (Figure 11) (ISO 23036-1:2021, Part I) [222], Codex-recommended pepsin digestion (ISO 23036-1:2021, Part II) [223,224], spectral imaging [225,226], electromagnetic parasite detection [227] and molecular analysis [223,228,229]. The effectiveness of the methods differs as well as their suitability for industrial application [228]. Nevertheless, even when the parasite is detected, there is still a risk of allergens remaining in the food. A concerning fact is that the heat- and pepsin-resistant allergen of *A. simplex*, Ani s 4, has been detected in commercial flour used in the production of fish and chicken feed, confirming the allergen transfer to fishmeal [206]. In view of this, a safe alternative, especially for people with Anisakis-induced allergies, is to eat fish from aquaculture, which has been proven to be free of zoonotic parasites, at least in the Mediterranean region [230].

## 7. Conclusions and Future Perspectives

A current challenge for public health is the fact that true burden of anisakidosis in most countries is still poorly estimated or unknown because epidemiological data are mostly of low quality. Furthermore, there are no standardised procedures between different laboratories and methods needed to confirm anisakidosis, without clear algorithms that would help clinicians diagnose and treat patients with anisakiasis. Therefore, anisakidosis remains a globally underestimated zoonosis, calling for improving disease surveillance and control to reduce morbidity and related costs for health systems. In view of this, future research should focus on the development of standardised procedures and, in particular, rapid diagnostic tests that could lead to a correct diagnosis in the shortest possible time. Although vaccines are the best preventive measure, it is unlikely that research on anisakidosis will go in this direction, considering that the disease does not currently represent a comparable burden to other parasitoses, especially neglected tropical diseases, for which vaccines have not yet been developed. Nevertheless, progress can and should be made in the development of measures to detect and eliminate infective larvae that are feasible in industrial environments in order to eliminate the risks to consumers at the fish processing stage before the products reach the market. Most countries need a framework for the prevention of anisakiasis as public health issue. Health organisations and policy makers can focus on new strategic control measures, launching public health educational campaigns for the general public about preventive measures together with campaigns to raise awareness of health professionals about anisakidosis. All these initiatives can be useful in gaining extended disease knowledge. Implementing One Health approach principles with different activities is important because promoting cross-professional collaboration is fundamental to understanding anisakidosis. Such an approach can build capacity for future research addressing the sensitisation of the general or professionally exposed population to parasites, in relation to the incidence of clinical anisakidosis cases and their impact on public health.

## 8. Literature Searching Criteria

This literature review was conducted using a methodology that focused on scientific articles in English, published in peer-reviewed journals. For the section on food safety considerations and prevention of anisakidosis, data were obtained within the decade from Scopus using the following search criteria: Title, Abstract, Keyword; anisakis AND viability OR biocidal activity. For other sections, data were obtained from Web of Science and PubMed without time restriction using the following criteria: Title, Abstract, Author Keywords (WoS) and MeSH terms (PubMed); anisakis OR anisakis simplex OR anisakis pegreffii OR anisakid* OR pseudoterranova OR pseudoterranova decipiens OR pseudoterranova azarasi OR pseudoterranova cattani OR phocanema OR contracaecum OR contracaecum osculatum OR anisakiasis OR anisakidosis OT pseudoterranovosis and combining all the sets. Finally, for seroepidemiological data, a more stringent strategy was applied using the following criteria and timeframe of five years (since the last published systematic review): Title, Abstract, Author Keywords (WoS) and MeSH terms (PubMed); (anisakis OR anisakid*) AND (sensitization OR sensitivity OR hypersensitivity OR allergy OR allergic reaction OR immunization OR immunoblot* OR skin prick test OR ELISA OR Enzyme-Linked Immunosorbent Assay OR IgE OR immunoglobulin E).

## Figures and Tables

**Table 1:** List of major *Anisakis* spp. allergens.

Allergen	Nematode Antigen	Protein	Reactivity in Sensitised Patients (%)	Reference
Ani s 1	Excretory–secretory (ES) product	Kunitz serine protease inhibitor	85	[160]
Ani s 2	Somatic	Paramyosin	88	[163]
Ani s 3	Somatic	Tropomyosin	Unknown	[164]
Ani s 4	ES	Cysteine protease inhibitor (cystatin)	27	[165]
Ani s 5	ES	SXP/RAL-2 family protein	25–49	[166]
Ani s 6	ES	Serin protease inhibitor (serpin)	18	[166]
Ani s 7	ES	Glycoprotein	83–100	[167]
Ani s 8	ES	SXP/RAL-2 family protein	25	[168]
Ani s 9	ES	SXP/RAL-2 family protein	13	[169]
Ani s 10	Unknown	Unknown	39	[170]
Ani s 11	Unknown	Unknown	47	[171]
Ani s 12	Unknown	Unknown	57	[171]
Ani s 13	Somatic	Haemoglobin	64.3–80.9	[172]
Ani s 14	Unknown	Unknown third-stage larval protein	53.8	[173]

**Table 2:** Thermal conditions and times required for inactivation of *Anisakis* spp. and *Phocanema* spp. in fisheries’ products.

Parasite	Recommended Thermal Treatment	Reference
*Anisakis simplex* s. l.	−20 °C for no less 24 h, in all parts of the product	[207]
	−35 °C for no less 15 h, in all parts of the product	[207]
	−20 °C for 7 days	[208]
	−35 °C for 15 h or until solid	[208]
	>60 °C for no less than 10 min	[105]
	90 °C for 10 min (same as for pathogenic bacteria)	[208]
*Phocanema decipiens* s. l.	−20 °C for 7 days	[208]
	−35 °C for 15 h or until solid	[208]
	60 °C for 10 min (3 cm-thick fillets) 70 °C for 7 min (3 cm-thick fillets) 10–12 min of cooking for each inch (~2.5) of thickness	[211]

## Appendix A

**Table A1:** The paratenic hosts of nematodes of *Anisakis* spp. recognised by molecular/genetic markers. The table also includes species formerly included in the genus *Anisakis* and now classified in the genus *Skrjabinisakis*. Note that *Anisakis* spp. may have been reported in other cephalopod/fish species but without molecular/genetic identification of the species. The taxonomy of cephalopods and fish listed in the table is based on the World Register of Marine Species (WoRMS, https://www.marinespecies.org/index.php (accessed on 15 September 2024)).

Host Species	*Anisakis* Species
Cephalopods	
Histioteuthidae	
*Histioteuthis bonnellii*	*Skrjabinisakis (Anisakis) physeteris* [231]
Sepiidae	
*Sepia officinalis*	*A. simplex* (s. s.) [232]
Ommastrephidae	
*Dosidicus gigas*	*S. physeteris* [233]
*Todarodes angolensis*	*A. pegreffii* [234]
*Todaropsis eblanae*	*A. simplex* (s. s.) [232,234], *A. pegreffii* [234]
*Todarodes sagittatus*	*A. simplex* (s. s.) [232], *A. pegreffii* [235], *S. physeteris* [236]
*Todarodes pacificus*	*A. typica* [237]
*Nototodarus sloanii*	*A. pegreffii*
*Illex coindettii*	*A. simplex* (s. s.) [232], *A. pegreffii* [238], *S. physeteris* [239]
Onychotheutidae	
*Ancistroteuthis lichtensteinii*	*S. physeteris* [13]
*Moroteuthis ingens*	*A. ziphidarum* [240]
Chondrichthyes	
Carcharinidae	
*Carcharinus brevipinna*	*A. typica* [49]
Osteichthyes	
Anoplogastridae	
*Anoplogaster cornuta*	*S. paggiae* [241]
Anoplopomatidae	
*Anopoploma fimbria*	*A. simplex* (s. s.), *A. berlandi* [242]
Belonidae	
*Belone belone*	*A. simplex* (s. s.) [232], *A. pegreffii* [232,243]
Berycidae	
*Beryx splendens*	*S. physeteris*, *S. brevispiculata*, *S. paggiae* [244]
Bothidae	
*Arnoglossus laterna*	*A. simplex* (s. s.) [245]
*Arnoglossus imperialias*	*A. pegreffii* [245]
Bramidae	
*Brama brama*	*A. pegreffii* [234]
Caesionidae	
*Caesio cuning*	*A. typica* [246]
Carangidae	
*Decapterus macarellus*	*A. typica* [247]
*Decapterus maruadsi*	*A. pegreffii* [248]
*Pseudocaranx dentex*	*A. pegreffii* [249]
*Selar crumenophthalmus*	*A. typica* [246,247]
*Seriola dumerili*	*A. pegreffii* [52]
*Seriola lalandi*	*A. pegreffii* [248]
*Trachurus capensis*	*A. pegreffii* [234]
*Trachurus declivis*	*A. pegreffii* [250]
*Trachurus mediterraneus*	*A. pegreffii* [251]
*Trachurus picturatus*	*A. simplex* (s. s.) [252,253], *A. pegreffii* [251,252], *A. typica* [252,253]
*Trachurus trachurus*	*A. simplex* (s. s.) [232], *A. pegreffii* [251], *A. typica* [254], *S. physeteris* [251,254,255]
Centracanthidae	
*Spicara smaris*	*A. pegreffii* [256]
Centrolophidae	
*Hyperoglyphe antarctica*	*A. berlandi* [250]
Citharidae	
*Citharus linguatula*	*A. simplex* (s. s.) [245], *A. pegreffii* [245]
Clupeidae	
*Alosa alosa*	*A. simplex* (s. s.), *A pegreffii* [257]
*Alosa fallax*	*A. simplex* (s. s.), *A pegreffii* [257]
*Clupanodon punctatus*	*A. simplex* (s. s.) [258], *A. pegreffii* [248]
*Clupea harengus*	*A. pegreffii* [234]
*Clupea pallasi*	*A. pegreffii* [259]
*Sardina pilchardus*	*A. pegreffii* [251,260]
*Sardinops sagax*	*A. simplex* (s. s.), *A. pegreffii*, *A. berlandi* [261]
*Sardinella zunasi*	*A. pegreffii* [248]
Congridae	
*Conger conger*	*A. simplex* (s. s.) [232], *A. pegreffii* [234]
*Conger myriaster*	*A. pegreffii* [259], *A. typica* [262]
Coryphaenidae	
*Coryphaena hippurus*	*A. simplex* (s. s.) [26], *A. pegreffii* [248], *A. typica* [252]
Cottidae	
*Myoxocephalus scorpius*	*A. simplex* (s. s.) [13]
Dussumeiriidae	
*Etrumeus whiteheadi*	*A. pegreffii* [234]
Emmelichthydae	
*Emmelichthys nitidus nitidus*	*A. pegreffii* [234]
Engraulidae	
*Engraulis encrasicolus*	*A. pegreffii* [251,263]
*Engraulis japonicus*	*A. pegreffii* [248]
Exocoetidae	
*Cheilopogon agoo*	*A. pegreffii* [248]
Gadidae	
*Boreogadus saida*	*A. simplex* (s. s.) [236]
*Gadus chalcogramma*	*A. simplex* (s. s.) [234,258], *A. pegreffii* [264], *S. brevispiculata*, *S. paggiae* [264]
*Gadus macrocephalus*	*A. pegreffii* [248]
*Gadus morhua*	*A. simplex* (s. s.) [242,246,265,266]
*Melanogrammus aeglefinus*	*A. simplex* (s. s.) [246,266]
*Micromestius poutassou*	*A. simplex* (s. s.) [232], *A. pegreffii* [232,235,251], *A. typica* [243], *S. physeteris* [251,255]
*Pollachius virens*	*A. simplex* (s. s.) [267]
*Trisopterus luscus*	*A. simplex* (s. s.) [232]
*Trisopterus minutus*	*A. zphidarum* [268]
Gempylidae	
*Rexea solandri*	*A. pegreffii*, *A. berlandi* [250]
*Thyrsites atun*	*A. pegreffii*, *A. berlandi* [258]
Gerreidae	
*Gerres oblongus*	*A. typica* [247]
Hexagrammidae	
*Hexagrammos agrammus*	*A. pegreffii*, *A. typica* [248]
*Hexagrammos otakii*	*A. pegreffii* [248]
*Pleurogrammus azonus*	*A. simplex* (s. s.) [269]
Lateolabracidae	
*Lateolabrax japonicus*	*A. pegreffii* [248]
Lotidae	
*Molva dypterygia*	*A. simplex* (s. s.) [232]
*Brosme brosme*	*A. simplex* (s. s.) [258]
Lophiidae	
*Lophius budegassa*	*A. simplex* (s. s.), *A. pegreffii* [13,270], *S. physeteris* [270]
*Lophius litulon*	*A. pegreffii* [248]
*Lophius piscatorius*	*A. simplex* (s. s.) [232], *A. pegreffii* [234], *S. physeteris* [270]
*Lophius vomerinus*	*A. pegreffii* [234]
Lutjanidae	
*Pinjalo lewisi*	*A. typica* [247]
*Pinjalo pinjalo*	*A. typica* [247]
Macrouridae	
*Macrourus berglax*	*A. simplex* (s. s.) [271]
Merluccidae	
*Macruronus novaezelandiae*	*A. pegreffii*, *A. berlandi* [250]
*Merluccius capensis*	*A. pegreffii* [258]
*Merluccius hubbsi*	*A. pegreffii* [258]
*Merluccius merluccius*	*A. simplex* (s. s.) [232,258], *A. pegreffii* [251,258], *A. typica* [235,258], *A. ziphidarum*, *S. physeteris*, *S. brevispiculata*, *S. paggiae* [254]
Monacanthidae	
*Thamnaconus modestus*	*A. pegreffii* [248]
Moridae	
*Mora moro*	*A. pegreffii*, *A. berlandi* [250]
*Pseudophycis bachus*	*A. pegreffii*, *A. berlandi* [250,258]
Moronidae	
*Dicentrarchus labrax*	*A. simplex* (s. s.) [272]
Mullidae	
*Mullus barbatus*	*A. simplex* (s. s.) [256]
*Mullus surmuletus*	*A. pegreffii* [251]
Muraenesocidae	
*Muraenesox cinereus*	*A. pegreffii* [248]
Murenidae	
*Muraena helena*	*A. pegreffii* [235]
Myctophidae	
*Electrona carlsbergi*	*A. berlandi* [273]
*Gymnoscopelus nicholsi*	*A. pegreffii* [273], *A. berlandi* [273]
*Myctophum punctatum*	*A. simplex* (s. s.), *A. pegreffii* [274]
Nemipteridae	
*Nemipterus bathybius*	*A. typica* [275]
*Nemipterus virgatus*	*A. typica* [275]
Ophidiidae	
*Genypterus capensis*	*A. pegreffii* [234]
Oreosomatidae	
*Allocyttus niger*	*A. berlandi* [13]
*Pseudocyttus maculatus*	*A. berlnadi* [13]
Osmeridae	
*Hypomesus japonicus*	*A. simplex* (s. s.) [269]
Paralichthydae	
*Pseudorhombus cinnamoneus*	*A. pegreffii* [276]
Pholidae	
*Pholis nebulosa*	*A. pegreffii* [248]
Phycidae	
*Phycis belennoides*	*A. pegreffii*, *S. physeteris* [26,235]
*Phycis phycis*	*A. pegreffii*, *A. typica*, *S. physeteris* [26,235]
Pinguipedidae	
*Parapercis colias*	*A. pegreffii*, *A. berlandi* [258]
Platycephalidae	
*Cociella crocodilus*	*A. pegreffii* [248]
*Platycephalus richardsoni*	*A. pegreffii* [277]
Pleuronectidae	
*Cleisthenes herzensteini*	*A. pegreffii* [248]
*Hippoglossoides dubius*	*A. pegreffii* [278]
*Hippoglossus hippoglossus*	*A. simplex* (s. s.) [236]
*Platichthys flesus*	*A. typica* [245]
*Pleuronectes platessa*	*A. simplex* (s. s.) [279]
*Pseudopleuronectes yokohamae*	*A. pegreffii* [248]
Pomacentridae	
*Lutjanus erythropterus*	*A. pegreffii* [248]
Rachycentridae	
*Rachycentron canadum*	*A. pegreffii* [248]
Salmonidae	
*Oncorhynchus gorbusha*	*A. simplex* (s. s.) [280]
*Oncorhynchus keta*	*A. simplex* (s. s.) [280]
*Oncorhynchus nerka*	*A. simplex* (s. s.) [280]
*Salmo salar*	*A. simplex* (s. s.) [258]
Scophtalmidae	
*Lepidorhombus boscii*	*A. sipmlex* (s. s.) [232], *A. pegreffii* [245]
Scienidae	
*Collichthys niveatus*	*A. pegreffii* [248]
*Larimichthys polyactis*	*A. pegreffii* [248]
*Nibea albiflora*	*A. pegreffii* [248]
*Pennahaia argentata*	*A. pegreffii* [248]
Scomberesocidae	
*Scomberesox saurus*	*A. simplex* (s. s.) [234]
Scombridae	
*Auxis thazard*	*A. pegreffii*, *A. typica* [248], *S. physeteris* [281]
*Euthynnus affinis*	*A. typica* [252]
*Euthynnus alletteratus*	*A. simplex* (s. s.), *A. pegreffii* [282]
*Katsuwonus pelamis*	*A. typica* [246]
*Rastelliger kanagurta*	Unpublished (GenBank accession AB432909)
*Sarda orientalis*	*A. typica* [252]
*Scomber australasicus*	*A. simplex* (s. s.), *A. pegreffii* [52,278], *A. typica*, *S. physeteris*, *S. brevispiculata*, *S. paggiae* [283]
*Scomber colias*	*A. pegreffii* [234,256]
*Scomber japonicus*	*A. simplex* (s. s.) [269,282], *A. pegreffii* [248,282,284], *A. ziphidarum*, *A. nascettii*, *A. typica*, *S. physeteris* [38,253]
*Scomber scombrus*	*A. simplex* (s. s.) [232], *A. pegreffii* [251], *A. typica*, *A. ziphidarum*, *A. nascettii* [282], *S. physeteris* [235]
*Scomberomorus commerson*	*A. typica* [252]
*Scomberomorus maculatus*	*A. typica* [247]
*Scomberomorus niphonius*	*A. pegreffii* [248]
*Thunnus albacares*	*A. typica* [247]
*Thunnus thynnus*	*A. simplex* (s. s.) [234], *A. pegreffii* [234,235], *A. typica* [254]
Scorpenidae	
*Helicolenus dactylopterus*	*A. pegreffii* [234]
*Hoplosebastes armatus*	*A. simplex* (s. s.), *A. pegreffii* [232]
*Scorpaena scrofa*	*A. simplex* (s. s.), *A. pegreffii* [232]
Sebastidae	
*Sebastolobus alascanus*	*A. berlandi* [246]
*Sebastodes fuscescens*	*A. pegreffii* [248]
Sillaginidae	
*Sillago sihama*	*A. pegreffii* [248]
Soleidae	
*Discoglossa cuneata*	*A. pegreffii* [245]
*Solea senegalensis*	*A. simplex* (s. s.) [245]
Sparidae	
*Diplodus annularis*	*A. pegreffii* [256]
*Pagellus bogaraveo*	*A. simplex* (s. s.), *A. pegreffii*, *A. typica*, *A. ziphidarum*, *S. physeteris* [285]
*Spondyliosoma cantharus*	*A. simplex* (s. s.) [232]
Sternoptychidae	
*Maurolicus muelleri*	*A. simplex* (s. s.) [286]
Stromateidae	
*Pampus argenteus*	*A. pegreffii* [248]
Synanceiidae	
*Inimicus japonicus*	*A. pegreffii* [248]
Synodontidae	
*Saurida elongata*	*A. pegreffii* [248]
Tetraodontidae	
*Takifugu niphobles*	*A. pegreffii* [248]
*Takifugu poecilonotus*	*A. pegreffii* [278]
Trachichthydae	
*Hoplostethus atlanticus*	*A. pegreffii* [234], *A. berlandi* [250]
*Hoplostethus cadenati*	*A. ziphidarum*, *A. typica*, *S. physteris*, *S. brevispiculata* [287]
*Hoplostethus mediterraneus*	*A. berlandi* [234]
Trachinidae	
*Echiichthys vipera*	*A. pegreffii* [235]
*Trachinus draco*	*A. pegreffii* [235]
Trichiuridae	
*Aphanopus carbo*	*A. simplex* (s. s.) [246], *A. pegreffii* [253], *A. ziphidarum*, *A. nascettii* [253], *S. physeteris* [236], *S. brevispiculata* [13], *S. paggiae* [288]
*Lepidopus caudatus*	*A. pegreffii* [258], *A. ziphidarum* [13], *S. physeteris* [13]
*Trichiurus lepturus*	*A. simplex* (s. s.) [13] *A. pegreffii* [235,248], *A. typica* [246]
Trichodontidae	
*Arctoscopus japonicus*	*A. pegreffii* [278]
Triglidae	
*Chelidonichthys kumu*	*A. pegreffii*, *A. typica* [248]
*Eutrigla gurnardus*	*A. simplex* (s. s.) [232]
Xiphiidae	
*Xiphias gladius*	*A. simplex* (s. s.) [289], *A. pegreffii* [234], *A. ziphidarum*, *A. typica* [254], *S. physeteris*, *S. brevispiculata*, *S. paggiae* [289]
Zeidae	
*Zeus faber*	*A. pegreffii* [251]
*Zeus japonicus*	*A. pegreffii* [248]
Zoarcidae	
*Zoarces elongatus*	*A. pegreffii* [248]

**Table A2:** Paratenic hosts of nematodes of *Phocanema* spp. recognised by molecular/genetic markers. Species included in table were formerly included in genus Pseudoterranova. Note that Phocanema spp. may have been reported in other cephalopod/fish species but without molecular/genetic identification of species. Taxonomy of cephalopods and fish listed in table is based on World Register of Marine Species (WoRMS, https://www.marinespecies.org/index.php (accessed on 15 September 2024)).

Host Species	*Phocanema* Species
Arhynchobatidae	
*Sympterygia bonapartii*	*Ph. cattani* [290]
*Atlantoraja castelanui*	*Ph. cattani* [290]
Channichthydae	
*Chaenocephalus aceratus*	*Ph. decipiens* E [291]
Cottidae	
*Myoxocephalus scorpius*	*Ph. decipiens* (s. s.) [54]
*Myoxocephalus quadricornis*	*Ph. bulbosum* [63], *Ph. azarasi* [26]
Gadidae	
*Gadus morhua macrocephalus*	*Ph. decipiens* (s. s.) [64], *Ph. bulbosum* [63], *Ph. azarasi* [63,64]
*Gadus morhua*	*Ph. krabbei* [54,292], *Ph. decipiens* (s. s.) [54,292], *Ph. bulbosum* [292]
*Gadus ogac*	*Ph. decipiens* (s. s.) [54]
*Pollachius virens*	*Ph. krabbei*, *Ph. decipiens* (s. s.) [54]
*Melanogrammus aeglefinus*	*Ph. krabbei* [54]
*Boreogadus saida*	*Ph. decipiens* (s. s.) [54]
Gempylidae	
*Thyrsites atun*	*Ph. cattani* [293]
Lotidae	
*Brosme brosme*	*Ph. decipiens* (s. s.) [54]
Macrouirdae	
*Macrourus berglax*	*Ph. bulbosum* [294]
Merluccidae	
*Merluccius gayi*	*Ph. cattani* [293]
Notothenidae	
*Notothenia coriiceps*	*Ph. decipiens* E [295]
*Notothenia neglecta*	*Ph. decipiens* E [26]
*Trematomus newnesi*	*Ph. decipiens* E [26]
Ophidiidae	
*Genypterus maculatus*	*Ph. cattani* [296]
Osmeridae	
*Osmerus eperlanus*	*Ph. decipiens* (s. s.) [291,297]
Paralichthydae	
*Paralichthys isosceles*	*Ph. cattani* [59]
*Paralichthys microps*	*Ph. cattani* [296]
*Paralichthys patagonicus*	*Ph. cattani* [295]
Percophidae	
*Percophis brasiliensis*	*Ph. cattani* [59]
Pinguipedidae	
*Pseudopercis semifasciata*	*Ph. cattani* [295]
Pleuronectidae	
*Hippoglossus hippoglossus*	*Ph. bulbosum*, *Ph. azarasi* [63]
*Hippoglosoides platessoides*	*Ph. decipiens* (s. s.) [54,292], *Ph. bulbosum*, *Ph. krabbei* [54]
*Reinherdtius hippoglossoides*	*Ph. bulbosum* [54]
Scombridae	
*Scombrus scombrus*	*Ph. decipiens* (s. s.) [292]
Scophthalmidae	
*Psetta maxima*	*Ph. krabbei* [54]
Serranidae	
*Acanthistius patachonicus*	*Ph. cattani* [295]
Triglidae	
*Prionotus nudigula*	*Ph. cattani* [59]

**Table A3:** Paratenic hosts of nematodes of *Contracaecum* spp. recognised by molecular/genetic markers. Note that *Contracaecum* spp. may have been reported in other cephalopod/fish species but without molecular/genetic identification of species. Taxonomy of cephalopods and fish listed in table is based on World Register of Marine Species (WoRMS, https://www.marinespecies.org/index.php (accessed on 15 September 2024)).

Host Species	*Contracaecum* Species
Bathydragonidae	
*Gymnodraco acuticeps*	*C. osculatum* D [258], *C. osculatum* E [26]
*Cygnodraco mawsonii*	*C. osculatum* D, *C. osculatum* E [26]
Channichthydae	
*Cryodraco antarcticus*	*C. osculatum* D [69,258], *C. radiatum* [298]
*Chionodraco hamatus*	*C. osculatum* D [69,258], *C. osculatum* [298]
*Pagetopsis macropterus*	*C. osculatum* D, *C. osculatum* E [26]
*Chaenodraco wilsoni*	*C. osculatum* D, *C. osculatum* E [26]
Cottidae	
*Myoxocephalus quadricornis*	*C. osculatum* (s. s.) [291], *C. osculatum* B [299]
Gadidae	
*Gadus chalcogrammus*	*C. osculatum* A [63,64]
*Gadus morhua macrocephalus*	*C. osculatum* A [63]
*Gadus morhua*	*C. osculatum* B [258]
Notothenidae	
*Notothenia neglecta*	*C. osculatum* D, *C. osculatum* E [69]
*Trematomus bernachii*	*C. osculatum* D [300], *C. osculatum* E [258]
*Trematomus hansoni*	*C. osculatum* D, *C. osculatum* E [300]
*Trematomus newneso*	*C*, *osculatum* D, *C. osculatum* E [300]
*Trematomus pennelli*	*C. osculatum* D, *C. osculatum* E [69]
Osmeridae	
*Mallostus vilosus*	*C. osculatum* B [299]
Pleuronectidae	
*Hippoglossus hippoglossus*	*C. osculatum* A [63]
*Hippoglossoides platessoides*	*C. osculatum* A, *C. osculatum* B [299]
*Pseudopleuronectes americanus*	*C. osculatum* B [299]
*Reinhardtius hippoglossoides*	*C. osculatum* A, *C. osculatum* B, *C. osculatum* (s. s.) [294]
